# Urine organic acid metabolomic profiling by gas chromatography mass spectrometry: Assessment of solvent extract evaporation parameters on the recovery of key diagnostic metabolites

**DOI:** 10.1016/j.cca.2024.120015

**Published:** 2025-01-15

**Authors:** Rachel S. Carling, Karolina Witek, Erin C Emmett, Claire Gallagher, Stuart J. Moat

**Affiliations:** a GKT School Medical Education, Kings College London, Strand, London WC2R 2LS, UK; b Biochemical Sciences, Synnovis, Guys & St Thomas’ NHSFT, London, UK; c Department of Medical Biochemistry, Immunology & Toxicology, University Hospital Wales, Cardiff CF14 4XW, UK; d School of Medicine, Cardiff University, University Hospital Wales, Cardiff CF14 4XN, UK

**Keywords:** Organic acids, Metabolomics, GC–MS, Inherited metabolic disease, Analysis

## Abstract

**Background:**

Analysis of urinary organic acids (UOAs) by gas chromatography mass-spectrometry (GC–MS) is widely used in metabolomic studies. It is a complex test with many limitations and pitfalls yet there is limited evidence in the literature to support best practice. This study investigated the impact of drying down time and temperature on the recovery of 16 key analytes from solvent extracts.

**Methods:**

Pooled urine specimens were enriched with organic acids. Urine aliquots (n = 3) were acidified and extracted into diethyl ether and ethyl acetate. Extracts were dried under nitrogen at ambient temperature (25 °C); 40 °C; 60 °C then left for 0; +5; +15 min. Dried extracts were derivatised with N,O,-bis-(trimethylsilyl)trifluoroacetamide prior to analysis by GC–MS. Urine specimens from individuals with biotinidase deficiency, maple syrup urine disease (MSUD) and ketotic hypoglycemia were analysed to demonstrate the potential clinical impact.

**Results:**

Recovery of shorter chain hydroxycarboxylic acids decreased significantly when extracts were dried above 25 °C (mean recovery 89 % at 60 °C, p < 0.01) or left under nitrogen post-drying (mean recovery at ambient + 15 min, 40 °C + 15mins and 60 °C + 15mins was 56 %, 12 % and 2 %, respectively, p < 0.01). Whilst dicarboxylic acids/medium chain fatty acids were unaffected by temperature (mean recovery 100 %), prolonged drying reduced recovery (mean recovery 85 % at 60 °C + 15mins, p < 0.01).

**Conclusions:**

Evaporation of solvent extracts with heat and/or prolonged drying under nitrogen results in significant losses of the shorter chain hydroxycarboxylic acids. The evaporation protocol must be carefully controlled to ensure accurate and reproducible results, preventing misdiagnoses and/or misinterpretation of results.

## Highlights

• Analysis of urine organic acids by GC–MS is widely used in clinical laboratories and increasingly, in metabolomics.
• Whilst this powerful technique can detect a wide range of compounds, it is not optimised for individual analytes.
• Without careful control (time/temperature) of the solvent evaporation step, hydroxycarboxylic acids can be lost.
• Potential for missed diagnosis of IMDs by clinical labs, and the collection of inaccurate data during metabolic studies.

## 1 Introduction

Urine organic acid analysis (UOA) using gas chromatography mass-spectrometry (GC–MS) has the capability to simultaneously detect ∼ 250 compounds in human urine and has therefore been utilised for metabolomic studies [1], [2], [3], [4], [5]. This technique, first described in 1980, [6] has been widely adopted worldwide as a first line investigation for inherited metabolic disease (IMD) as it can enable the diagnosis of ∼ 150 disorders [5], [7]. However, it should be recognised that whilst UOA can identify a wide range of compounds, it is not optimised for the detection of any of them and is essentially a screening method. UOA is a complex process, prone to many limitations and pitfalls [7], [8], [9] and as there are currently no commercially available reagent kits suitable for clinical application, services rely exclusively on laboratory developed tests with in-house reagents, standards and methodologies.

Whilst there are several publications documenting sample preparation protocols [4], [5], [6], [7], [8], [10] there is a lack of evidence to support best practice, and no consensus opinion on the merits of the different approaches [11]. These include the use of urease [12]; sodium sulphate [5], [6]; oximation (oximation stabilises α-keto acids and aldehydes) [13]; choice of extraction solvent(s) [6], [14], [15]; derivatisation reagent, time and temperature [16], [17], [18]; type of glassware used [19], all of which impact on results, as do any inconsistencies in what is a relatively complex, manual process. The presence of artefacts formed during the derivatisation process can result in multiple peaks for the same compound or unexpected components in the GC analysis, both of which can lead to difficulty in quantification and ambiguity in diagnosis [20], [21].

Similarly, there is also a paucity of evidence-based protocols for the separation and measurement of the derivatised products by GC–MS. During GC–MS analysis, thermal degradation of heat-labile components in the sample can occur at high temperatures such as those commonly found in the injection port; column; ion source and transfer line. This can result in the occurrence of multiple peaks for one compound in the GC chromatogram and/or degradation of compounds prior to GC–MS analysis [15]. Furthermore, analytes are often quantified using sub-optimal approaches; full scan rather than selective reaction monitoring; internal rather than external calibration; structural analogue internal standards rather than stable isotope labels (SIL); periodic calibration rather than real time; aqueous rather than urine-based calibrators. Thus, methods are neither harmonised nor standardised, making comparison of results between laboratories challenging.

Results from the European Research Network for evaluation and improvement of screening, Diagnosis and treatment of Inherited disorders of Metabolism (ERNDIM) Quantitative Urine Organic Acid external quality assessment (EQA) scheme, which has more than 120 participating laboratories, emphasise this point, with poor reproducibility and under-recovery of UOA acknowledged to be a significant problem [22]. For many IMDs, diagnosis is based upon the presence or absence of a key compound(s), so absolute accuracy is often viewed as less important. However, the large intra- and inter-laboratory variation observed for several key pathognomonic metabolites [22] are indicative of more fundamental errors such as inconsistent and/or under-recovery as opposed to simple measurement bias. Errors of this magnitude could result in failure to detect key analytes / compounds contributing to a missed diagnosis. Whilst poor performance can be explained by the analytical challenges outlined previously, these do not explain why the under-recovery of certain compounds is particularly poor. Inconsistent recovery of the low molecular weight (MW) hydroxycarboxylic acids has been noted in the authors’ laboratories and it was hypothesised that the loss of these volatile compounds was occurring during sample preparation, specifically, when the solvent extract is evaporated to dryness under nitrogen. However, again, there are no data in the literature to confirm or refute this theory.

The complexity of urinary organic acid analysis is the likely reason why many laboratories have not modified either the sample preparation procedure, or the GC–MS methodology, since the test was first introduced. As such, these legacy methods have not been updated to reflect developments in GC and MS technology, the increased availability of SILs, advances in spectral identification and the potential for automation. To achieve accreditation under ISO 15189:2022 standards, clinical laboratories must now be able to justify, and evidence, the clinical utility of a given test. Combined with the advantages of ensuring data generated by metabolomic studies is widely transferable, these are powerful drivers for improvements in current laboratory practice.

The current interest in metabolomics has resulted in numerous studies being undertaken, producing a wealth of data, the quality of which is rarely considered. If this quantitative data is to be translated into meaningful clinical practice, consideration must be given to analytical performance parameters such as accuracy, sensitivity and reproducibility. Therefore, the aim of this study is to investigate the impact of drying down time and temperature on the recovery of 16 key metabolites from solvent extracts of human urine.

## 2 Materials and methods

### 2.1 Chemicals and reagents

Ethyl acetate (AnalaR NORMAPUR) and diethyl ether (AnalaR NORMAPUR) were obtained from VWR (Leicestershire, UK). Pyridine (>99 %), 32 % HCL, NaCl (>99.5 %) and N,O,-bis-(trimethylsilyl)trifluoroacetamide (BSTFA) containing 1 % trimethyl-chlorosilane (TMCS) were obtained from Sigma (Dorset, UK). L-lactic acid sodium salt (>98 %), 2-hydroxybutyric sodium salt (>97 %), 3-hydroxypropionic acid (30 % solution in water), 3-hydroxybutyrate sodium salt (>99 %), 2-hydroxyisovaleric acid (99 %), acetoacetic acid lithium salt (>90), 3-hydroxyisovaleric acid (>95 %), 2-hydroxisocaproic acid (>99), methylmalonic acid (99 %), succinic acid (99 %), glutaric acid (99 %), adipic acid (99 %), 3-hydroxy-3-methylglutararic acid (>95 %), suberic acid (98 %) sebacic acid (99 %) and orotic acid (>98 %) were obtained from Sigma (Dorset, UK). Methyl-d3-malonic acid (99.8 %), uracil-d4 (>99 %) and sodium-L-Lactate-3,3,3-d3 (99.8 %) were obtained from QMX (Thaxted, UK). (2H6)-3-hydroxyisovaleric acid (>99 %) was obtained from VU Medical (Amsterdam, Netherlands).

### 2.2 Standards and internal quality control (IQC)

Individual stock standards (20 mL, 10 mM) and SIL standards (10 mL, 10 mM) were prepared in distilled water. Aliquots of both standards and SILs were stored at −70 °C prior to use. Mixed SIL stock standard was prepared by addition of 1 mL of each stock standard before making to 100 mL with water.

Two levels of lyophilised, freeze-dried IQC material (IQCS Organic acids) were obtained from SKML (MCA labs, Netherlands). IQC levels 1 and 2 were enriched with orotic acid stock standard, 0.4 mL and 1.2 mL (2.5 mM), respectively to give final concentrations of 10 µmol/L and 30 µmol/L. ICQ materials and patient specimens were aliquoted and stored at −20 °C prior to use.

### 2.3 Sample preparation

Sample preparation and analysis was based on standard procedures described elsewhere [6], [7], [9], [10], [11]. In brief, the volume of urine extracted is corrected for the creatinine concentration to ensure that a ‘standardised’ amount of organic acids are extracted from each sample, thus minimising the ‘dilution-effect’ and enabling consistent interpretation and quantification of organic acids [23]. Mixed internal standard (50 µL) is added to the urine followed by approx. 1 g of sodium chloride. The sample is acidified with HCl (1 M, 20 µL) prior to sequential liquid–liquid extraction with ethyl acetate (2.5 mL) and diethyl ether (2.5 mL). The two organic phases are combined and evaporated to dryness under nitrogen (25 mL/min) at ambient temperature. The residue is reconstituted in pyridine (20 µL) and BSTFA containing 1 % TMCS (75 µL) and heated at 75˚C for 30 min.

### 2.4 Analysis of samples by gas chromatography mass spectrometry (GC–MS)

Chromatographic separation was performed on a Shimadzu QP 2010 ULTRA GC (Shimadzu. Milton Keynes, UK) coupled to a non-polar (HP-5 ms Ultra) fused silica capillary column (30 m x 0.25 x 0.25 um) (Agilent, Didcot, UK). Sample (1 µL) was injected with a split ratio 20:1 at 300 °C; flow rate of helium carrier gas was 30.7 mL/min. The GC oven temperature program started at 80° (5 min), followed by a linear gradient of 8° per min to 280 °C, then holding constant for 10 min. The electron ionisation source was at 70 eV and the transfer line temperature was 280 °C. Data were acquired in full scan mode (*m*/*z* 50–550). The method described is in routine use at the author’s (RSC’s) laboratory and is accredited to ISO 15189:2022 standard.

### 2.5 Study design

Urine (50 mL) was collected from three healthy volunteers after informed consent had been obtained, in line with local governance protocols. Each urine pool was enriched with the organic acid stock standards (500 µL, 10 mmol/L) to give final concentrations of 100 µmol/L. Residual urine specimens received in the laboratory for routine analysis of organic acids were also used. These were from patients with biotinidase deficiency (n = 1), MSUD (n = 1) and ketotic hypoglycemia (n = 1) respectively.

Urinary creatinine measurement was performed using an enzymatic method (creatinase) on a Roche Cobas 701 analyser (Roche Diagnostics Ltd, West Sussex, UK). The creatinine concentration was used to determine the volume of water required to dilute the urine sample to 1 mmol/L equivalent concentration [23]. Prior to analysis, samples were stored at −20 °C.

The effect of drying down temperature (ambient, 40 °C, 60 °C) on the recovery of 16 metabolites was assessed by replicate measurements of the enriched pools of urine (n = 3). The metabolites were categorised based on functional group. Group 1 contained seven hydroxycarboxylic acids and one monocarboxylic acid, and Group 2 contained four dicarboxylic acids, three medium chain fatty acids and one pyrimidine monocarboxylic acid. Multiple aliquots (n = 30) of each enriched urine pool were taken through the first stage of the sample preparation process: following addition of SIL mixed stock, each aliquot was acidified, and the liquid–liquid extraction performed. The solvent phases (n = 30) from each individual urine pool were then combined and mixed gently, effectively producing three solvent pools, each derived from one of the three enriched urine pools. Each solvent pool was aliquoted (3 mL) into n = 27 glass tubes (Chromacol 13 mm screw vial, round bottom, borosilicate glass (type 1), ThermoScientific, Loughborough, UK) and the contents evaporated under nitrogen (flow rate 25 mL/min) at varying conditions; set A (n = 3) were evaporated at ambient temperature (25˚C), removed and capped immediately upon reaching dryness; set B (n = 3) were evaporated to dryness at ambient temperature, left for a further 5 mins, then removed and capped; set C (n = 3) were evaporated at ambient temperature, left for a further 15 mins, then removed and capped; set D (n = 3) were evaporated at 40˚C, removed and capped immediately upon reaching dryness; set E (n = 3) were evaporated at 40˚C, left for a further 5 mins, then removed and capped; set F (n = 3) were evaporated at 40˚C, left for a further 15 mins, then removed and capped; set G (n = 3) were evaporated at 60˚C, removed and capped immediately upon reaching dryness; set H (n = 3) were evaporated at 60˚C, left for a further 5 mins, then removed and capped; set I (n = 3) were evaporated at 60˚C, left for a further15 mins, then removed and capped.

All sample sets were then derivatised into the trimethylsilyl esters prior to analysis by GC–MS.

Recovery of the sixteen metabolites was determined for sample sets B to I, relative to sample set A, which acted as the control. For each compound of interest, the mean (SD) peak area of the characteristic ion was determined for each sample set and compared with the mean peak area for that compound in sample set A. Characteristic ions (*m*/*z*) for each compound were as follows: 2-hydroxypropionic acid (lactic acid) 191, 2-hydroxybutyric acid 131, 3-hydroxypropionic acid 219, 3-hydroxybutyric acid 233, 2-hydroxyisovaleric acid 145, acetoacetic acid 231, 3-hydroxyisovaleric acid 131, methylmalonic acid 247, 2-hydroxyisocaproic acid 159, succinic acid 247, glutaric acid 261, adipic acid 275, 3-hydroxy-3-methylglutaric acid 273, suberic acid 303, orotic acid 254 and sebacic acid 303.

Data sets generated were processed using Microsoft-Excel 2016. A T-test was used to determine whether there was a significant difference in the mean recoveries of group 1 and 2 compounds for each sample set (p < 0.01).

The effect of drying down temperature (ambient, 40 °C, 60 °C) on three diagnostic patient specimens was also assessed. Each specimen was analysed in triplicate, with one solvent extract evaporated at ambient temperature and removed immediately upon reaching dryness, one solvent extract evaporated at 40˚C and left for a further 15 mins, and one solvent extract evaporated at 60˚C and left for a further 15 min. Following derivatisation and analysis by GC–MS, the extracted ion current chromatograms for each specimen were compared qualitatively.

## 3 Results

**Fig. 1:**
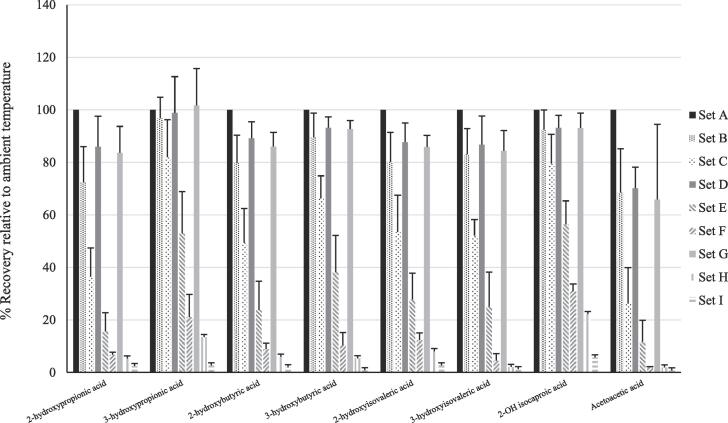
Comparison of recoveries of the 8 compounds in Group 1, following evaporation under nitrogen at different temperatures and times.

**Fig. 2:**
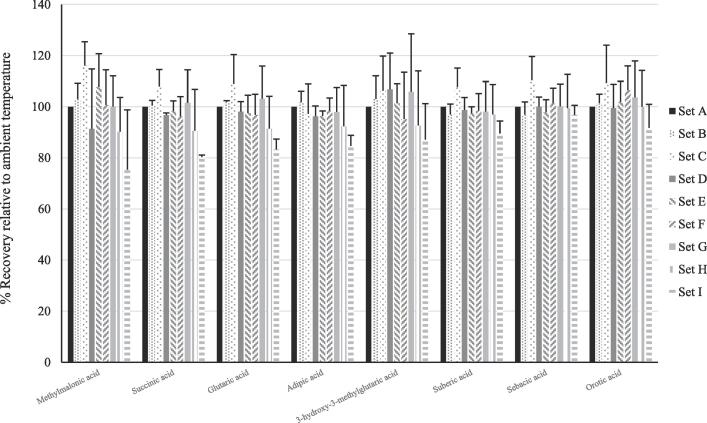
Comparison of recoveries of the 8 compounds in Group 2, following evaporation under nitrogen at different temperatures and times.

Mean recoveries of the 16 compounds in sample sets B to I are summarised in Table 1. The mean recovery of group 1 compounds was significantly lower than that of the group 2 compounds (p < 0.01) for all sample sets (B to I). Mean recovery for group 1 and 2 compounds across all sample sets was 46 % (range 2 – 88) and 98 % (range 86 – 108) respectively. The relative recoveries for each metabolite in samples sets B to I are summarised in Fig. 1, Fig. 2. There were significant differences (p < 0.01) in the mean recovery of group 1 and 2 metabolites for all sample sets.

**Table 1:** Table 1: Mean recovery of the 16 compounds in sample sets B to I.

	**Experiment**	**A**	**B**	**C**	**D**	**E**	**F**	**G**	**H**	**I**
		**Ambient**	**Ambient + 5 mins**	**Ambient + 15 mins**	**40 °C**	**40 °C + 5 mins**	**40 °C + 15 mins**	**60 °C**	**60 °C + 5 mins**	**60 °C + 15 mins**
	**Metabolite**	**Mean (SD) % analyte recovery**								
**Group 1**	2-Hydroxypropionic acid	100	72 (14)	37 (11)	86 (12)	16 (7)	7 (1)	84 (10)	5 (3)	2 (0)
	2-Hydroxybutyric acid	100	80 (11)	49 (13)	89 (6)	24 (11)	9 (2)	86 (5)	6 (2)	2 (0
	3-Hydroxypropionic acid	100	97 (8)	82 (14)	99 (14)	53 (16)	21(9)	102(14)	13 (8)	3 (1)
	3-Hydroxybutyric acid	100	89 (9)	66 (9)	93 (4)	38 (14)	10 (5)	93 (3)	5 (3)	1 (0)
	2-Hydroxyisovaleric acid	100	80 (11)	53 (14)	88 (7)	28 (10)	12 (3)	86 (4)	8 (3)	3 (1)
	Acetoacetic acid	100	68 (17)	26 (14)	70 (27)	12 (8)	2 (0)	66 (29)	2 (1)	1 (1)
	3-Hydroxyisovaleric acid	100	83 (10)	52 (6)	87 (11)	25 (13)	5 (3)	84 (8)	2 (1)	1 (1)
	2-hydroxyisocaproic acid	100	92 (8)	80 (11)	93 (5)	56 (9)	31(3)	93 (6)	22 (4)	6 (1)
	**All group 1 analytes**	100	83 (9)	56 (18)	88 (8)	31 (15)	12 (9)	87 (10)	8 (6)	2 (2)
**Group 2**	Methylmalonic acid	100	103 (6)	116 (9)	91 (23)	108 (13)	101(6)	100 (14)	90 (12)	75 (13)
	Succinic acid	100	100 (2)	108 (7)	97 (1)	98 (4)	96 (4)	102 (8)	91 (13)	80 (16)
	Glutaric acid	100	102 (0)	109 (11)	98 (4)	98 (7)	97 (6)	103 (8)	91 (13)	83 (13)
	Adipic acid	100	102 (4)	97 (12)	96 (4)	96 (2)	98 (1)	98 (5)	92 (10)	85 (16)
	3-hydroxy-3-methylglutaric acid	100	103 (9)	106 (14)	107 (14)	101(8)	95 (8)	106 (18)	93 (23)	87 (21)
	Suberic acid	100	97 (4)	108 (7)	99 (5)	97 (3)	98 (2)	98 (7)	97 (12)	89 (12)
	Sebacic acid	100	97 (5)	110 (9)	100 (4)	98 (5)	101 (3)	100 (6)	99 (9)	97 (13)
	Orotic acid	100	101(3)	109 (15)	100 (9)	102 (8)	106 (10)	104 (9)	100 (14)	92 (14)
	**All group 2 analytes**	100	101(2)	108 (5)	98 (4)	100 (4)	99 (3)	101 (3)	94 (4)	86 (6)

**Fig. 3:**
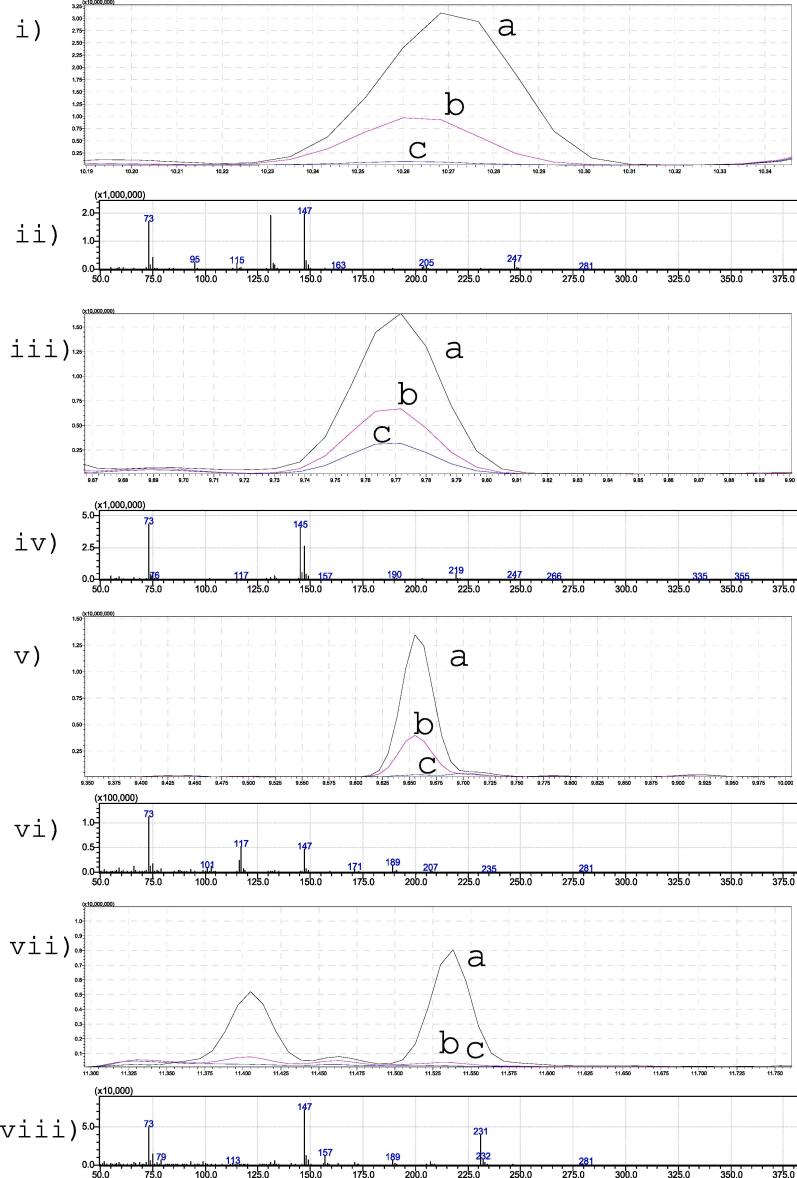
Total ion current chromatograms from three diagnostic urine specimens showing the reduction in peak abundance of four key metabolites when solvent extracts are evaporated at higher temperatures thus illustrating the potential for a missed diagnosis. Figure 3i is from an individual with biotinidase deficiency and shows the peak abundance of 3-hydroxyisovalerate when the solvent extract is evaporated at a) ambient temperature, b) 40˚C plus 15 mins and c) 60˚C plus 15 mins. Figure 3iii is from an individual with MSUD and shows the reduction in peak abundance of 2-hydroxyisovalerate when the solvent extract is evaporated at a) ambient temperature, b) 40˚C plus 15 mins and c) 60˚C plus 15 mins. Figure 3v and 3vii are from an individual with ketotic hypoglycemia and shows the reduction in peak abundance of 3-hydroxybutyrate and acetoacetate respectively, when the solvent extract is evaporated at a) ambient temperature, b) 40˚C plus 15 mins and c) 60˚C plus 15 mins. The corresponding mass spectra for 3-hydroxyisovalerate, 2-hydroxyisovalerate, 3-hydroxybutyrate and acetoacetate are shown in figures 3ii, iv, vi and viii respectively.

Qualitative comparison of the chromatograms from the three diagnostic urine specimens demonstrated significant losses of the key metabolites as the time and temperature of the solvent evaporation increased. The reduction in the peak abundance of each key metabolite was approximated relative to that in the solvent extract evaporated at ambient temperature. In the specimen from the individual with biotinidase deficiency, the decrease in abundance of 3-hydroxyisovalerate was approximately 70 % in the extract evaporated at 40˚C + 15 min, and 98 % in the extract evaporated at 60˚C + 15 min. In the specimen from the individual with MSUD, the decrease in abundance of 2-hydroxyisovalerate was approximately 60 % in the extract evaporated at 40˚C + 15 min, and 81 % in the extract evaporated at 60˚C + 15 min. In the specimen from the individual with ketotic hypoglycemia, the decrease in abundance of 3-hydroxybutyrate and acetoacetate was approximately 82 and 97 % respectively in the extract evaporated at 40˚C + 15 min, and 99 % for both compounds in the extract evaporated at 60˚C + 15 min. These findings are comparable with those seen for sample sets B to I. See Fig. 3 and supplementary material S1.

## 4 Discussion

To our knowledge, this is the most comprehensive study performed assessing the impact of drying down temperature and drying time on the recovery of analytes from solvent extracts. Significant differences in the recovery of metabolites between groups 1 and 2 are observed. The metabolites in group 2 comprised of dicarboxylic acids, medium chain fatty acids (C6–C10) and orotic acid, a pyrimidine monocarboxylic acid, whereas the group 1 metabolites are all hydroxycarboxylic acids with the exception of acetoacetic acid, a monocarboxylic acid. It is therefore unsurprising that the shorter chain, more volatile group 1 metabolites were prone to significant losses compared with the group 2 metabolites, where under-recovery was only evident after over-drying for 15 mins at 60˚C.

These data suggest that boiling point/volatility may not be the only factor contributing to under-recovery. Even at ambient temperature, significant under-recovery of the group 1 metabolites occurred if they were left under nitrogen for 15 min post-evaporation. It is hypothesised that this could be due to adsorption of small hydroxy acids to the surface of the glass tube which has been described previously [19] and may be a variable effect; both tube size and volume of extract being contributing factors. Glass tubes are often washed and re-used. Cleaning procedures vary (i.e. acid wash, alkaline detergent, solvent wash etc.) as does the quality of the glass tube in which to perform the solvent extraction. The recommended approach would be to use silylated glass tubes as this renders the glass surface less reactive and inhibits materials from adhering to the surface. However, many clinical laboratories deem the use of such tubes cost prohibitive.

Other factors which may be contributing to the variable intra-laboratory recovery of metabolites include differences in sample preparation i.e. oximation; use of urease; drying with anhydrous sodium sulphate; volume of urine extracted; size and type of glass tube; inconsistencies and efficacy of the solvent extraction step which is generally performed manually; and the nitrogen flow rate used to dry the extract.

The clinical implication of these findings is the potential for under-recovery of key diagnostic metabolites leading to misinterpretation of UOA profiles, resulting in inappropriate further investigation(s) and / or a missed diagnosis. See Fig. 3 and supplementary material S1. For example, the loss of volatile metabolites such as 3-hydroxybutyric acid and acetoacetic acid in relation to the more stable longer chain compounds (adipic, suberic and sebacic acid) could lead to interpretation of an inappropriate dicarboxylic aciduria in the absence of a ketotic response (see Figure S3) leading to unnecessary further investigations to exclude a fatty acid oxidation disorder. In IMDs where a low MW volatile compound accumulates, other increases of higher MW, less volatile compounds will also accumulate e.g. isovalerylglycine in isovaleric acidaemia or methylcitrate and propionylglycine in propionic acidaemia. However, poor recovery of low MW compounds could potentially lead to missed diagnosis of other IMDs where an increased excretion of low MW volatile compounds is the key diagnostic finding e.g. biotinidase deficiency (3-hydroxyisovaleric acid, Fig. 3i), MSUD (2-hydroxyisocaproic and 2-hydroxyisovaleric acid, Fig. 3iii). See also Supplementary material S1, Figures S1-S3.

By implementing simple, inexpensive changes to current practice, laboratories can improve the robustness of UOA analysis and avoid these pitfalls. Much of the detailed knowledge of UOA analysis is not well documented in the literature and therefore raising awareness amongst laboratory scientists of the complexities of the method and its limitations is an important first step which can be addressed through staff training and continuous competency assessment. Based on the evidence reported here, the Authors recommend standardising the drying down process at ambient temperature and ensuring tubes are removed from nitrogen as soon as dryness is reached. This is an important message to communicate to laboratory scientists because several of the commonly cited publications on organic acid methodology suggest solvent extracts are evaporated under nitrogen at 37˚C [10] or 60˚C [5], [14], or do not specify [3], [8]. Furthermore, implementation of two simple control measures designed to monitor critical steps in the process are also recommended; (1) Inclusion of IQC samples containing some, or all, of these volatile compounds with every batch of patient samples would enable identification of any gross error(s) affecting a given batch of samples however, it would not allow detection of error in an individual sample; (2) Inclusion of additional SILs of some of these key hydroxycarboxylic acids, e.g. d3-3-hydroxypropionic acid and d6-3-hydroxyisovaleric acid could, in principle, provide a check at an individual sample level. Monitoring of the peak area intensity of a characteristic ion(s) for each SIL in each sample would enable detection of a sample specific recovery issue. Given that several factors contribute to variation in peak area response, the utility of this approach would be improved by monitoring peak area intensity relative to an ion from a more stable compound. It is often commented on that the inclusion of additional SILs results in an increasingly complex UOA chromatogram; however, it is important for laboratories to challenge these statements, which are often ‘historic’ and based on older technology and take advantage of developments such as deconvolution software which solve these issues [24].

## 5 Conclusion

Optimisation of the sample preparation and legacy GC–MS methods, in combination with additional SILs and IQC monitoring will enable laboratories to improve the robustness and resilience of UOA measurements. Ensuring the quality of the data produced will be advantageous for both routine clinical analysis and metabolomics research.

This study provides evidence of the impact that the drying down conditions of the solvent extracts can have on the recovery of key organic acids and related compounds and highlights the potential for this to result in missed diagnoses and/or misinterpretation of results leading to unnecessary further investigations. There are several simple, inexpensive control measures that laboratories can implement to help identify such analyte losses. Future work is required to fully explore the automation of the sample preparation processes used for UOA by GC–MS.

## CRediT authorship contribution statement

**Rachel S. Carling:** Writing – original draft, Visualization, Supervision, Resources, Methodology, Formal analysis, Conceptualization. **Karolina Witek:** Writing – review & editing, Resources, Methodology, Investigation, Formal analysis, Data curation. **Erin C Emmett:** Writing – review & editing, Investigation. **Claire Gallagher:** Writing – review & editing, Investigation. **Stuart J. Moat:** Writing – original draft, Visualization.

## Funding

Funding to cover the cost of reagents and consumables was provided by the Synnovis’ Innovation Accelerator Fund. SJM and RSC are supported by 10.13039/100008392Medical Research Council grant number MR/Y008057/1.

## Declaration of competing interest

The authors declare that they have no known competing financial interests or personal relationships that could have appeared to influence the work reported in this paper.

## Data Availability

Data will be made available on request.
