# Supplementary material for: Urine organic acid metabolomic profiling by gas chromatography mass spectrometry: Assessment of solvent extract evaporation parameters on the recovery of key diagnostic metabolites
Source: Clin Chim Acta. 2025 Jan 15;565:120015. doi: 10.1016/j.cca.2024.120015 (PMC11876077; doi:10.1016/j.cca.2024.120015)
Supplement: Supplementary Data 1 [file mmc1.pptx]

## Slide 1
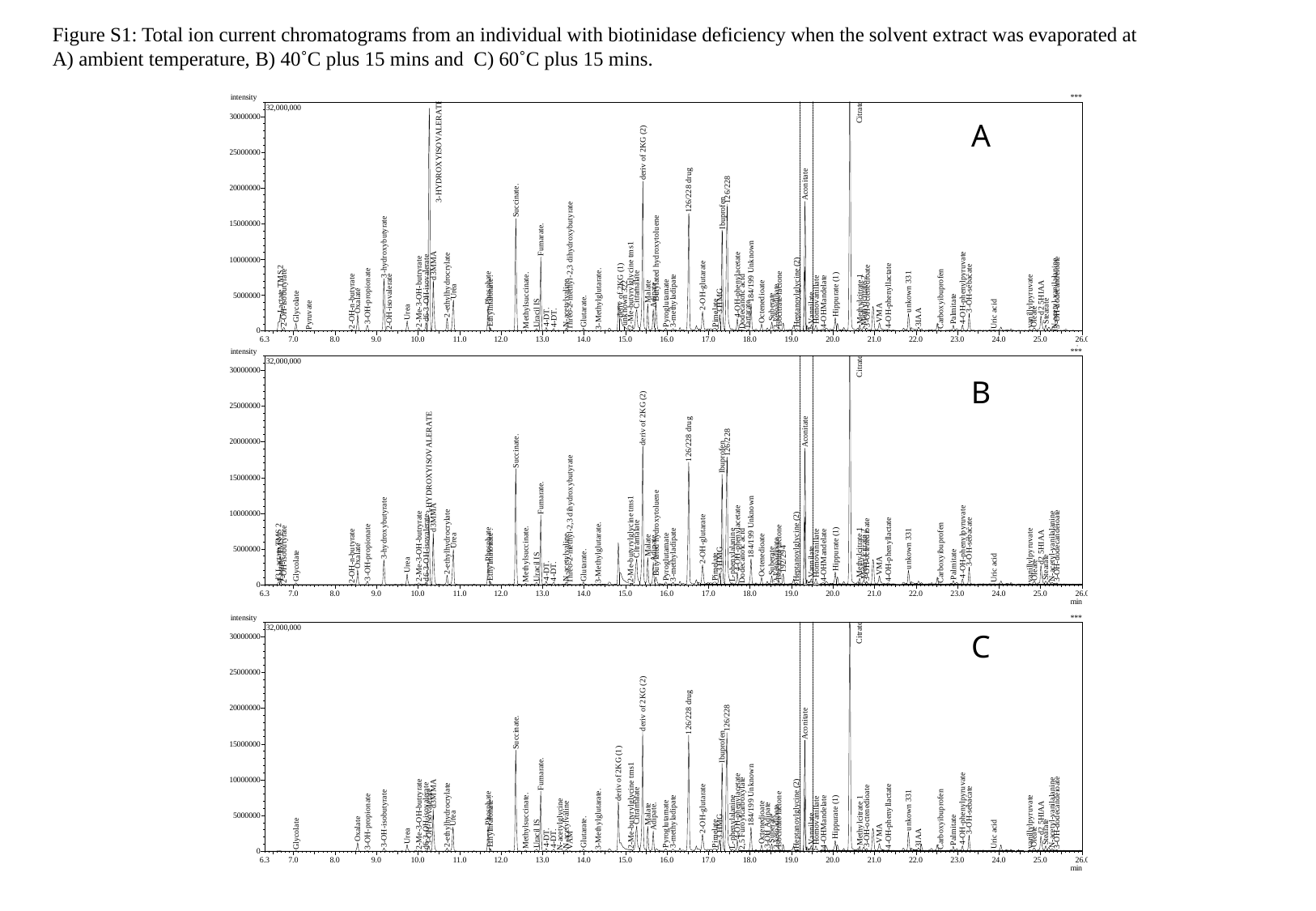

Figure S1: Total ion current chromatograms from an individual with biotinidase deficiency when the solvent extract was evaporated at A) ambient temperature, B) 40˚C plus 15 mins and C) 60˚C plus 15 mins.
A
B
C

## Slide 2
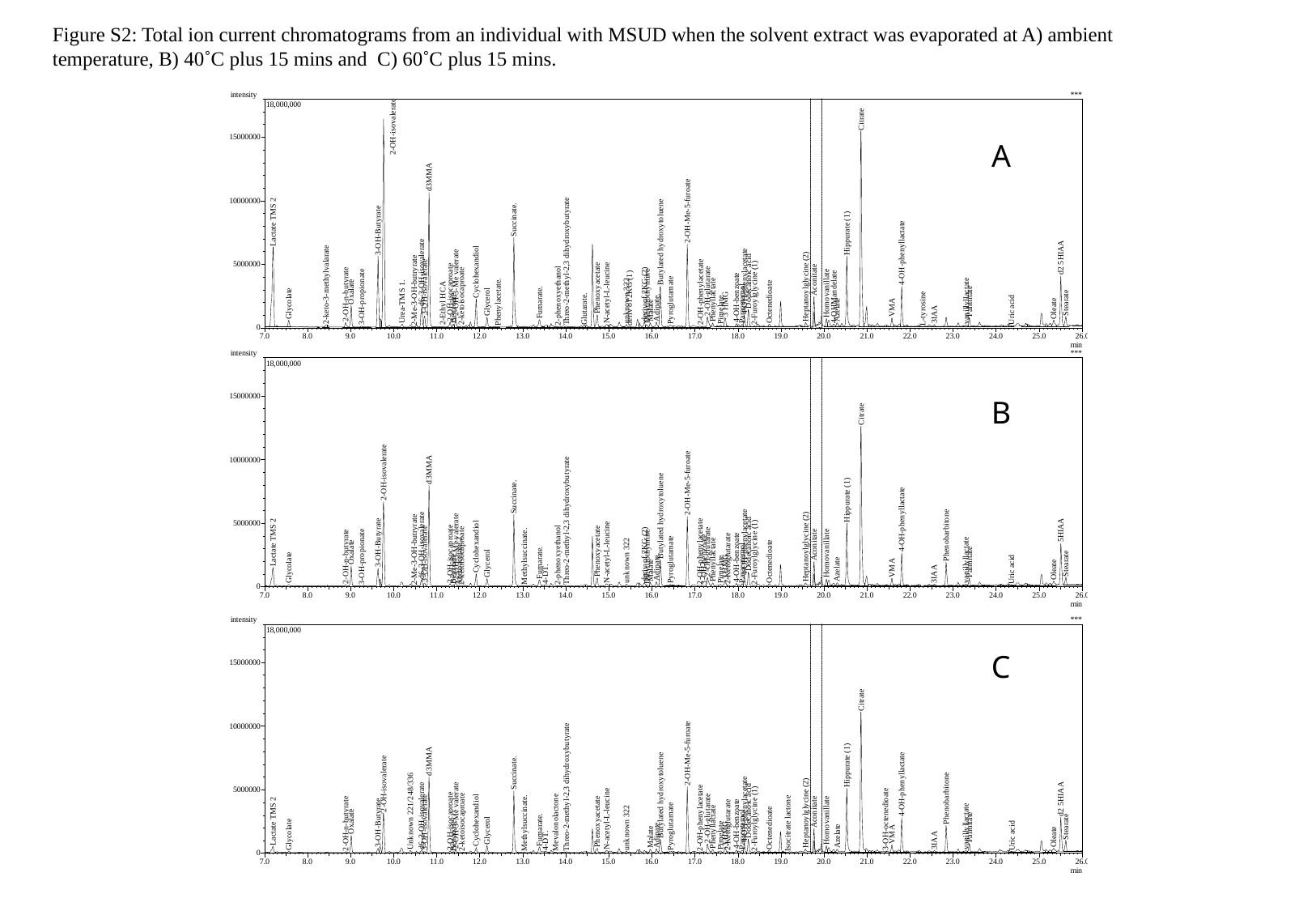

Figure S2: Total ion current chromatograms from an individual with MSUD when the solvent extract was evaporated at A) ambient temperature, B) 40˚C plus 15 mins and C) 60˚C plus 15 mins.
A
A
B
B
C
C

## Slide 3
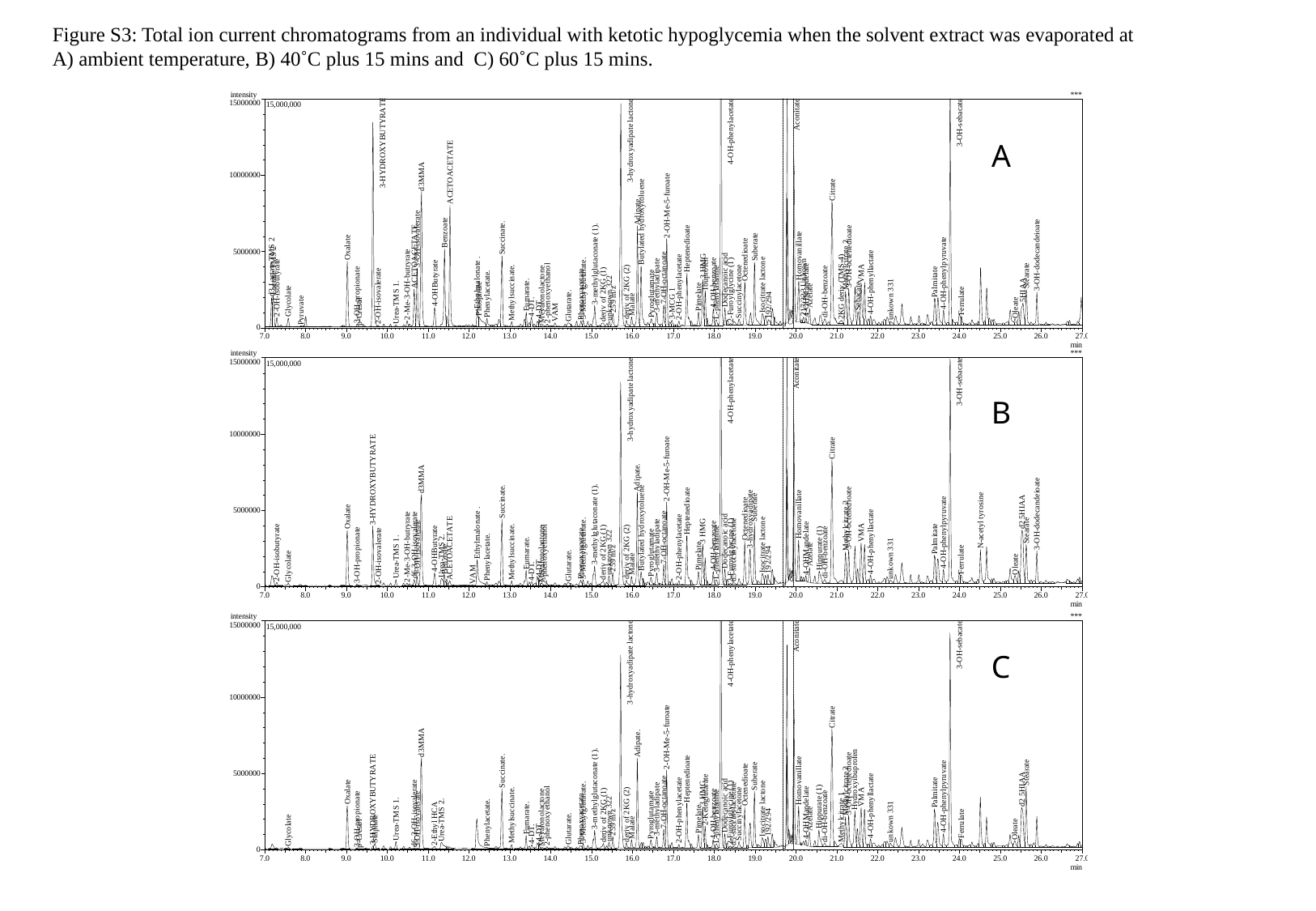

Figure S3: Total ion current chromatograms from an individual with ketotic hypoglycemia when the solvent extract was evaporated at A) ambient temperature, B) 40˚C plus 15 mins and C) 60˚C plus 15 mins.
A
A
B
B
C
C
